# Mitochondrial DNA: a molecular switch driving sterile neuroinflammation

**DOI:** 10.1186/s40035-026-00540-w

**Published:** 2026-02-13

**Authors:** Abhishek Jauhari, Tanisha Singh, Diane L. Carlisle, Robert M. Friedlander

**Affiliations:** Neuroapoptosis Laboratory, Department of Neurological Surgery, School of Medicine, University of Pittsburgh, Pittsburgh, PA USA

**Keywords:** Mitochondrial DNA, Mitochondria, Neuroinflammation, Neurodegeneration

## Abstract

Mitochondrial DNA (mtDNA) plays a pivotal role in the regulation of neuroinflammation, acting as a potent trigger of innate immune responses when released into the cytoplasm or extracellular space. mtDNA is structurally similar to bacterial DNA, containing unmethylated CpG motifs that are readily recognized by immune sensors. Under conditions of cellular stress, injury, or mitochondrial dysfunction, mtDNA can escape into the cytoplasm, where it activates the cGAS (cyclic GMP–AMP synthase)–STING (stimulator of interferon genes) signaling pathway, or it can be detected extracellularly by Toll-like receptors on immune cells. These signaling events lead to the production of pro-inflammatory cytokines and type I interferons, amplifying neuroinflammatory responses. In the central nervous system, this process contributes to the pathogenesis of various neurodegenerative and inflammatory conditions, such as Alzheimer’s disease (AD), Parkinson’s disease (PD), Huntington’s disease (HD), etc.. The dual role of mtDNA as both a damage-associated molecular pattern and a signaling molecule underscores its importance as a therapeutic target for modulating neuroinflammation and protecting against progressive neuronal damage. In this review, we will discuss the implications of mtDNA-mediated neuroinflammation in neurodegenerative diseases, including AD, PD, and HD, highlighting its potential as a diagnostic biomarker and therapeutic target.

## Introduction

Neuroinflammation is a common characteristic feature in neurodegenerative diseases (NDDs) and aging brains, which plays a major role in cognitive decline and neurological problems. NDDs such as Alzheimer’s disease (AD), Parkinson’s disease (PD), Huntington’s disease (HD), and amyotrophic lateral sclerosis (ALS) have all been associated with neuroinflammation, which further speeds up disease progression.

Microglia, the brain resident immune cells, play an important role in the regulation of neuroinflammation and synaptic degeneration. Microglial activation leads to both beneficial and harmful effects [1, 2]. In a healthy brain, microglia maintain a dynamic balance between protective, anti-inflammatory functions and detrimental, pro-inflammatory signaling [1, 3, 4]. However, in NDDs, this microglia balance is disrupted, and the activation of pro-inflammatory microglia contributes to neuronal damage and synaptic loss [5–7]. The inflammatory response amplifies through the release of cytokines, chemokines, and reactive oxygen species (ROS), creating a cycle of chronic inflammation that accelerates neurodegeneration and results in cognitive and motor impairments [8, 9].

Mitochondria, “powerhouses” of cells, are critical for neuronal survival due to their central role in energy production, calcium buffering, redox balance, and regulation of apoptotic pathways. Mitochondria contain their own DNA (mitochondrial DNA, mtDNA) in multiple copies and encode for 13 proteins required for oxidative phosphorylation (OXPHOS). mtDNA is vulnerable to oxidative damage due to its proximity to ROS production sites and the relatively lower capacity of DNA repair pathways. Unlike nuclear DNA, mtDNA lacks histones and is therefore more susceptible to oxidative damage, deletions, and point mutations. Accumulation of mtDNA damage could impair the OXPHOS system, leading to defects in electron transport chain (ETC) activity, increased ROS generation, and progressive bioenergetic failure, which contribute to the pathogenesis and progression of NDDs [10]. Further, mitochondrial structural damage compromises membrane integrity, which allows the release of mitochondrial contents such as mtDNA, cardiolipin, and mitochondrial proteins into the cytosol. The released mitochondrial content in cytosol or extracellular spaces acts as danger-associated molecular patterns (DAMP) and triggers innate immune responses. Cytosolic or extracellular mtDNA induces an innate immune response, which triggers neuroinflammation via immune sensors like the cyclic GMP–AMP synthase (cGAS)-stimulator of interferon genes (STING) pathway and Toll-like receptors (TLR) [11, 12]. Therefore, understanding the mtDNA-mediated neuroinflammation is crucial to develop targeted therapies for NDDs and the aging brain.

In recent years, substantial progress has been made to understand the role of neuroinflammation in the aging brain and NDDs; however, many questions remain unanswered. Specifically, the mechanisms that activate and regulate neuroinflammation and interplay between mtDNA, immune signaling, and neuroinflammatory pathways are not fully understood. This review aims to explore the emerging role of mtDNA as a neuroinflammatory switch. Here, we review the mechanisms of mtDNA release that triggers immune responses in the brain, and we highlight its potential as a therapeutic target in aging brain and NDDs. Understanding how mitochondrial dysfunction contributes to mtDNA release and subsequent neuroinflammation will open new avenues for therapeutic interventions aimed at mitigating neuroinflammation and its detrimental effects on brain health.

## mtDNA structure, maintenance, and vulnerability

In humans, mtDNA is a circular genome of 16,500 base pairs, which is located in many copies in the mitochondrial matrix [13]. mtDNA encodes a total of 37 genes, of which 13 code for proteins involved in OXPHOS, 22 code for tRNA, and 2 code for rRNA [14]. This distinct gene set on mtDNA enables mitochondria to have their own independent protein synthesis system, allowing them to carry out ATP synthesis efficiently. However, the structural, functional, and maintenance work of mitochondria are dependent on thousands of nuclear-encoded proteins. Neurons are among the most energy-demanding cells in the body, and their health and proper functioning depend on mitochondria to keep up with this constant demand. mtDNA is an important factor for OXPHOS and ATP production; therefore, it supports neuronal activity, synaptic transmission, and the maintenance of bioenergetic homeostasis [15]. Mitochondria synthesize ATP through OXPHOS, which involves the transfer of electrons through the ETC. However, during ETC, excessive ROS is generated, including superoxide anions and hydrogen peroxide. mtDNA proximity to these ROS production sites makes them more vulnerable to damage, mutations, deletions, and genetic instability [16]. This constant mtDNA exposure to ROS increases the oxidative damage probability, which accumulates over time, and leads to mitochondrial dysfunction [17, 18]. Mitochondria possess some DNA repair enzymes, such as mitochondrial polymerase gamma (POLG) and a version of base excision repair; however, these systems are not as efficient as those in the nucleus [19]. The combination of high exposure to ROS and inefficient repair systems results in a higher mutation rate in mtDNA and a decline of mitochondrial ability to produce energy [20]. Some mtDNA regions are more prone to mutations, particularly in genes encoding respiratory complex components. One of the most frequently observed mtDNA mutations is the large-scale 4977-bp deletion, also known as the “common deletion”, which spans several OXPHOS genes and is often flanked by direct repeats that predispose these regions to slipped-strand mispairing during replication [21, 22]. These deletions accumulate with age and have been observed at high levels in NDD [23–25]. mtDNA copy number is identified as a dynamic parameter that represents mitochondrial biogenesis and turnover. In aging brains and NDDs, mtDNA copy number alterations have been documented, which further compromise OXPHOS and respiratory capacity [26–30]. The functional consequences of accumulated mtDNA damage are profound, as oxidization, mutation, or deletion of mtDNA molecules can impair the assembly and function of respiratory chain complexes, leading to deficient ATP synthesis and increased ROS production [19]. Beyond the bioenergetic defects, damaged mtDNA may leak from mitochondria into the cytoplasm or extracellular space [31]. Such extranuclear mtDNA acts as a potent DAMP, capable of activating innate immune sensors including cGAS, TLR9, and the NOD, LRR and pyrin domain-containing protein 3 (NLRP3) inflammasome, thus triggering neuroinflammatory cascades [32–34]. Neurons, defined by their high metabolic demand, long lifespan, and limited regenerative capacity, are especially vulnerable to mitochondrial dysfunction. Accumulation of mtDNA damage is particularly detrimental and has been implicated in the pathogenesis of NDDs such as AD, PD, HD, and ALS. Over time, progressive mtDNA mutations compromise mitochondrial integrity, leading to increased neuroinflammation, synaptic dysfunction, and neuronal loss. This vicious cycle of mtDNA damage accumulation and mitochondrial dysfunction further accelerates neurodegeneration and disease progression.

## Mechanisms of mtDNA release

**Fig. 1:**
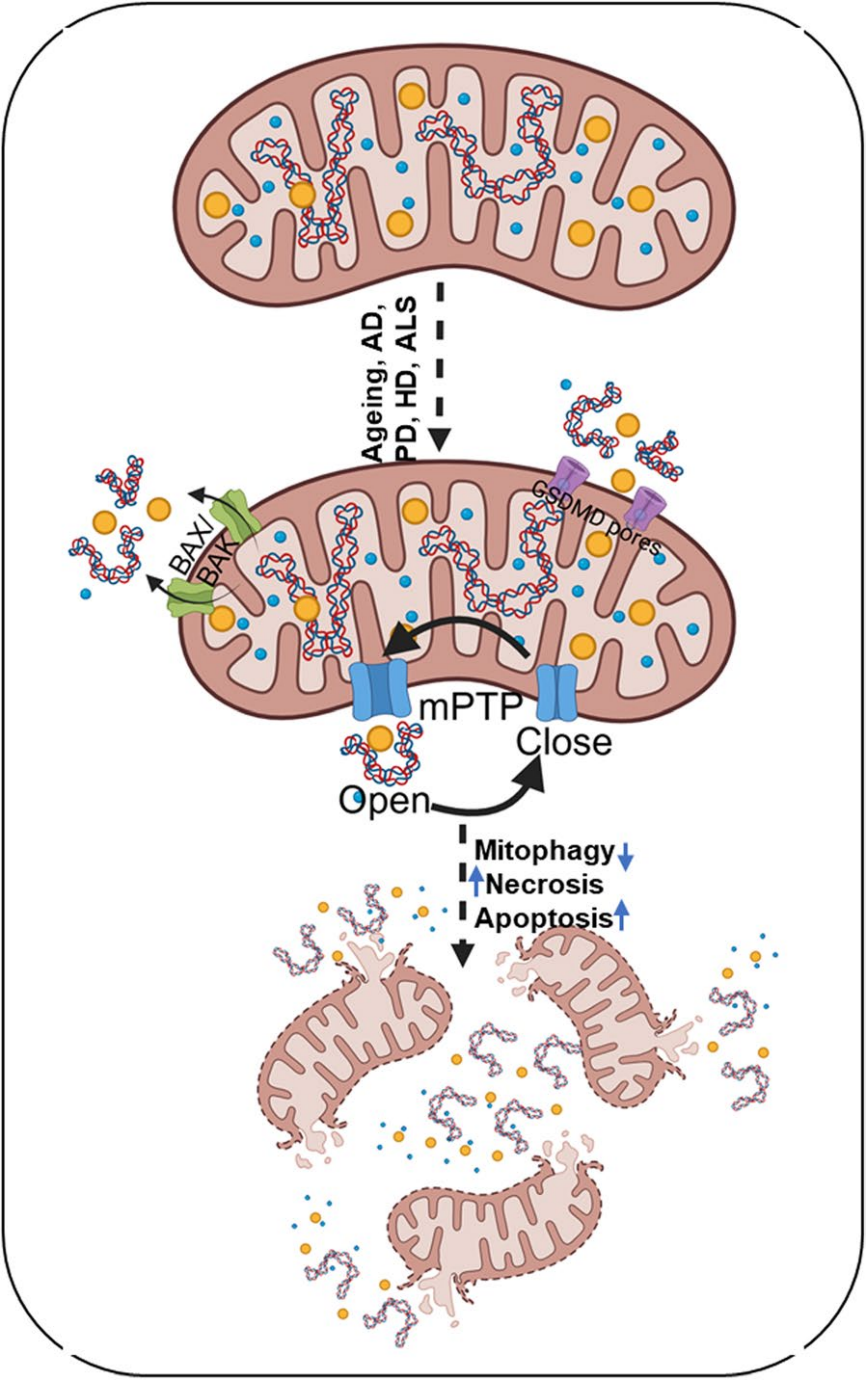
Schematic illustration depicting major cellular pathways that promote mtDNA escape from mitochondria into the cytosol and extracellular space under neurodegenerative conditions. Neurodegenerative conditions induce mitochondrial membrane permeabilization through BAX/BAK macropores, mitochondrial permeability transition pore (mPTP) opening, and Gasdermin (GSDMD) pores. Defective mitophagy, apoptosis, and necrosis further exacerbate mtDNA accumulation

The mtDNA is normally stored in the mitochondrial matrix, enclosed by the inner and outer mitochondrial membranes (OMM), protecting it from recognition by cellular immune sensors. Under physiological conditions, mitochondrial outer and inner membranes ensure that mtDNA primarily functions for mitochondrial gene expression and energy metabolism. However, during mitochondrial stress (physical, chemical, and biological) and damage, mtDNA could be abnormally released into the cytosol or extracellular space. This cytosolic or extracellular mtDNA functions as a potential DAMP, triggering innate immune responses and neuroinflammation. Diverse physical, chemical, and biological insults can trigger mtDNA release from mitochondria by different mechanisms (Fig. 1).

### Mitochondrial permeability transition pore (mPTP) opening and membrane rupture

mPTP is a multi-protein complex that forms a non-selective channel across the inner mitochondrial membrane [35]. When mPTP is open, it allows the passage of solutes ≤ 1.5 kDa, which leads to mitochondrial depolarization, swelling, rupture of the outer membrane, and release of material including mtDNA and cytochrome *c* [36]. mPTP opening can be caused by several factors, including higher mitochondrial Ca^2^⁺ level, increased oxidative stress, inorganic phosphate, and depletion of adenine nucleotide [35]. Opening of the mPTP plays a crucial role in cell death pathways, particularly in ischemia–reperfusion injury and different NDDs [37, 222]. Under pathological conditions such as calcium overload, higher ROS, ATP depletion, or ischemic injury, the mPTP can open transiently or persistently, leading to dissipation of the mitochondrial membrane potential, matrix swelling, and rupture of OMM [38–41]. This rupture of OMM permits the release of mitochondrial contents, which includes mtDNA fragments, into the cytosol, where they act as a potent DAMP [42, 43]. Although the precise molecular composition of the mPTP remains under investigation, cyclophilin D (CypD) is recognized as a key regulatory component [44, 45]. Pharmacological inhibition of CypD by cyclosporin A or genetic deletion reduces mPTP opening, mtDNA release, and downstream inflammation in various disease models [46–48]. In neurons, mPTP-mediated mtDNA release has been implicated in excitotoxicity and neuroinflammation, linking mitochondrial dysfunction to neurodegenerative pathology [12, 49].

In NDDs, persistent mPTP opening is increasingly recognized as a common mechanism driving neuronal injury [50]. In AD, Aβ accumulates in mitochondria and interacts with CypD, and promotes oxidative stress, collectively lowering the calcium threshold for mPTP opening. CypD deletion or inhibition rescued mitochondrial function and cognitive performance in murine models [51]. In PD, mitochondrial toxins like MPTP (1-methyl-4-phenyl-1,2,3,6-tetrahydropyridine) and rotenone induce oxidative stress and calcium overload, while α-synuclein (α-syn) accumulates at mitochondria and induces mPTP opening [52]. In HD, mutant Huntingtin (mHTT) disrupts calcium levels and increases mitochondrial ROS, lowering the threshold for calcium-induced mPTP opening in mitochondria [53, 54]. Together, these studies establish a common pathological cascade in NDDs. Disease-specific proteins (Aβ, α-syn, mHTT) induce mitochondrial calcium overload and oxidative stress, which sensitize the mPTP, leading to mitochondrial failure, mtDNA release, and neuronal degeneration.

### Dysfunctional mitophagy and impaired mitochondrial quality control

Mitochondria maintain their functional integrity through a coordinated quality control network, which includes mitochondrial fission and fusion, proteolytic degradation of oxidized/damaged proteins, and the removal and/or recycle of dysfunctional mitochondria via mitophagy [55, 56]. Mitophagy is crucial for removing depolarized, dysfunctional, or oxidized mitochondria that contain oxidatively damaged mtDNA [57, 58]. The canonical PINK1–Parkin-mediated mitophagy enables the recognition and ubiquitination of damaged mitochondria, facilitating their autophagic clearance, while the receptor-mediated pathways involving BNIP3, NIX, and FUNDC1 provide additional layers of regulation, particularly under stress conditions relevant to aging and NDDs [59–62]. Compromised mitophagy, which is increasingly observed with aging and in NDDs, allows damaged mitochondria to accumulate, increasing the possibility of mtDNA instability and release [60, 63]. In microglia, age-associated decline in mitophagic efficiency exacerbates mitochondrial dysfunction, promoting excessive ROS production and calcium dysregulation. These conditions sensitize mitochondria to prolonged mPTP opening, leading to matrix swelling, rupture of the OMM, and subsequent release of mtDNA fragments into the cytosol [42, 45, 48]. The cytosolic mtDNA can then act as a potent DAMP, activating innate immune pathways and sustaining chronic neuroinflammatory signaling in disorders such as AD, PD and HD.

In NDDs, impaired mitophagy causes accumulation of defective mitochondria containing oxidatively damaged mtDNA, which, upon release, can activate innate immune sensors, like cGAS, and promote neuroinflammation [63, 64]. Notably, the presence of Aβ exacerbates mitochondrial ROS production and impairs mitophagy, further enhancing mitochondrial dysfunction [65, 66]. In PD, mutations in the *PINK1* and *Parkin* genes, which encode key regulatory proteins of mitophagy, prevent the efficient clearance of damaged mitochondria [60]. As a result, neurons accumulate damaged or dysfunctional mitochondria, leading to excessive ROS production, increased sensitivity to mPTP opening, and cytosolic release of mtDNA, which activates innate immune signaling in microglia and accelerates neurodegeneration [67]. Similarly, in HD, mHTT not only impairs autophagy initiation and mitochondrial trafficking, but also directly inhibits the TIM23 protein import complex, reducing the import of nuclear-encoded mitochondrial proteins and causing early mitochondrial dysfunction and oxidative stress [68, 69]. This combination of defective mitophagy and impaired protein import exacerbates the proteostatic stress, promotes aggregate formation, and further compromises mitochondrial dynamics. The released mtDNA can activate cGAS–STING signaling, creating a self-reinforcing loop where inflammation further impairs mitochondrial quality control, promotes additional mtDNA release, and accelerates neuronal degeneration. Collectively, these observations highlight a convergent pathogenic mechanism in NDDs, in which mitochondrial quality control failure, through impaired mitophagy, defective protein import, and ROS-driven dysfunction, links mitochondrial damage, mtDNA-mediated inflammation, and protein aggregation [68–73].

### Apoptosis and BAX/BAK-mediated outer membrane permeabilization

Mitochondria are known to be a center for neuronal survival and death, which integrates metabolic signals and stress responses to decide and activate apoptosis. In the intrinsic apoptotic pathway, the BCL-2 family proteins play a critical regulatory role. Pro-apoptotic effectors BAX and BAK oligomerize upon activation, inducing mitochondrial outer membrane permeabilization (MOMP), a crucial event during apoptosis [73, 74]. Traditionally, MOMP is recognized for cytosolic release of cytochrome *c*, Smac/DIABLO, and other intermembrane space proteins, thereby activating caspase cascades and executing apoptosis [75]. However, recent work has demonstrated that MOMP facilitates the mtDNA release into the cytosol [76, 77]. Mechanistically, BAX and BAK assemble into large, lipidic pores within the OMM that can dynamically enlarge over time [78]. During apoptosis, the BAX/BAK pores permit protrusion or “herniation” of the inner mitochondrial membrane, carrying mitochondrial matrix content, including mtDNA, into the cytosol [76]. This process effectively breaks the mitochondrial compartmentalization that normally keeps mtDNA away from innate immune sensors. Unlike transient mPTP observed during mitophagy, apoptotic MOMP often results in continuous membrane disruption, greatly increasing the possibility of mtDNA release. Importantly, even in the absence of full caspase activation, BAX/BAK-mediated membrane permeabilization can still cause mtDNA leakage and trigger inflammatory responses [79]. This phenomenon is particularly relevant in post-mitotic neurons, where caspase activation is often tightly controlled, yet sub-lethal mitochondrial permeabilization can still promote chronic inflammation.

In the context of NDDs, this mechanism has profound implications. In AD, Aβ accumulation within neurons increases mitochondrial ROS production and sensitizes mitochondria to apoptosis by promoting BAX activation [80]. The activated BAX forms large enough pores to release mtDNA into the cytosol, which then amplifies neuroinflammation through the cGAS–STING signaling. Similarly, in PD, α-syn aggregates localize to mitochondria, impair mitochondrial dynamics, and increase oxidative stress, which together trigger BAX/BAK-mediated MOMP [81]. In HD, mHTT impairs mitochondrial calcium buffering and promotes oxidative stress, facilitating BAX recruitment to mitochondria [82]. BAX/BAK pore formation in HD models can similarly enable mtDNA release, feeding into a cycle of inflammation and neurodegeneration. These insights reveal a dual pathogenic role of BAX/BAK-mediated MOMP in neurological diseases beyond executing apoptosis: it can directly promote mtDNA release, which can activate neuroinflammation and drive disease progression. This convergence of mitochondrial dysfunction, apoptosis, and innate immune activation highlights therapeutic opportunities. Strategies that stabilize mitochondrial membranes, modulate BAX/BAK pore dynamics [83], or block downstream mtDNA sensing pathways [84] could inhibit inflammation without fully blocking apoptosis.

### Necrosis, necroptosis, and pyroptosis, inflammatory cell death modalities

Unlike the controlled, typically non-inflammatory process of apoptosis, necrotic and necroptotic cell death are characterized by plasma membrane integrity loss, resulting in the extracellular release of intracellular components, including intact mitochondria or mitochondrial fragments that harbor mtDNA [85, 86]. Similarly, pyroptosis, a caspase-1-dependent form of cell death, induces gasdermin-D pore formation in the plasma membrane, culminating in cellular swelling and rupture [87, 88]. These lytic cell death pathways promote the release of DAMPs, notably mtDNA, into the extracellular milieu [33, 89]. Extracellular mtDNA could engage pattern recognition receptors (PRR) on microglia, astrocytes, and infiltrating immune cells, serving as a potent pro-inflammatory signal that drives neuroinflammation in acute brain injury, ischemia, and chronic NDDs.

### Extracellular vesicle (EV)-mediated secretion of mtDNA

Recent studies have revealed that cells actively secrete EVs, such as exosomes and microvesicles [90]. These EVs can encapsulate whole mitochondria, mitochondrial fragments, or naked mtDNA, shielding them from extracellular degradation and enabling intercellular communication [90]. Under conditions of cellular stress, neurons, astrocytes, and microglia have all been shown to release mtDNA-containing EVs [67, 91]. This EV-mediated transfer of mtDNA may amplify neuroinflammatory responses by activating PRR in recipient cells, thereby facilitating the spread of pathological signals across neural networks. Mechanistically, EV biogenesis depends on the endosomal sorting complexes required for transport machinery and is modulated by stress-responsive pathways, although the precise molecular mechanisms that govern selective packaging of mtDNA into EV remain incompletely understood [92].

## mtDNA: sensing and immune activation

Under physiological conditions, mtDNA is securely sequestered within the mitochondrial matrix. However, physical injury, oxidative stress, mPTP opening, or BAX/BAK-mediated membrane permeabilization can trigger its release into the cytosol or extracellular space [32, 76]. Once liberated, mtDNA is perceived as a foreign-like molecular signature, activating an evolutionarily conserved network of nucleic acid sensors such as cGAS–STING, NLRP3 inflammasome, and TLR9 [33]. This signaling cascade effectively links mitochondrial dysfunction to sterile inflammation.

In the central nervous system (CNS), these processes are especially consequential. Neurons and microglia can detect and sense cytosolic mtDNA, driving neuroinflammatory responses characterized by cytokine production, inflammasome activation, and recruitment of peripheral immune cells [12]. Dysregulated mtDNA-mediated signaling has emerged as a major contributor to NDD progression [12, 93–95]. Thus, mtDNA release acts as a molecular bridge coupling cellular stress to chronic neuroinflammation. The following sections examine the major mtDNA sensors and their downstream pathways that sculpt immune responses in the aging and diseased brains.

### cGAS–STING pathway—cytosolic DNA sensing

**Fig. 2:**
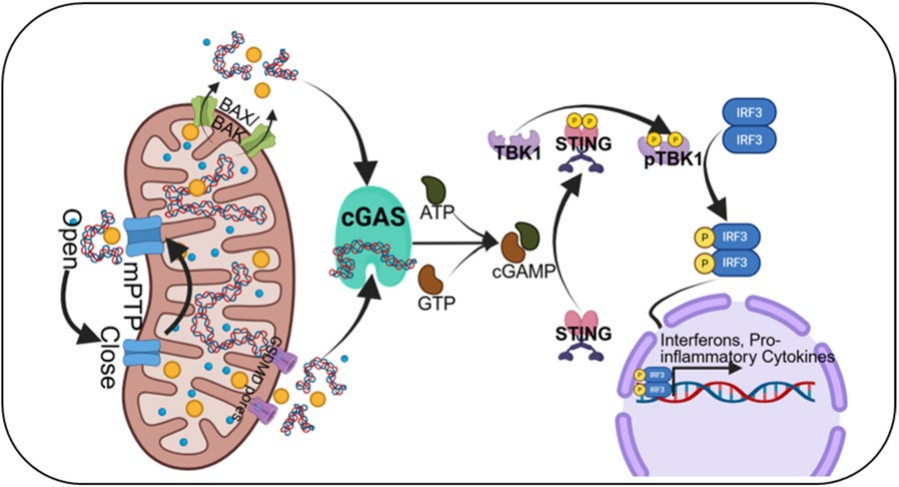
Schematic illustrating activation of the cGAS–STING pathway following the release of mitochondrial DNA (mtDNA) into the cytosol during cellular stress or mitochondrial dysfunction. Cytosolic mtDNA is sensed by cyclic cGAS, leading to the production of the second messenger cGAMP. cGAMP binds to and activates STING on the endoplasmic reticulum, triggering its translocation to the Golgi apparatus and subsequent recruitment of TBK1. This signaling cascade results in phosphorylation and nuclear translocation of IRF3, culminating in the induction of type I interferons and proinflammatory cytokines

The discovery of the cGAS–STING pathway has fundamentally reshaped our understanding of how cells detect cytosolic DNA as a danger signal, establishing a direct molecular link between mitochondrial injury and innate immunity [96–98]. Historically, innate DNA sensing was studied primarily in the context of viral infection; however, subsequent research demonstrated that endogenous nucleic acids, particularly mtDNA released under cellular stress, can also activate this pathway and trigger sterile inflammation [43]. Mechanistically, cGAS is a cytosolic sensor that binds dsDNA independent of sequence [96]. Upon dsDNA binding, cGAS catalyzes the synthesis of the second messenger cyclic GMP–AMP (cGAMP), which uniquely contains a mixed 2′–5′ and 3′–5′ phosphodiester linkage conferring high stability and potent signaling capacity [97]. cGAMP binds and activates the adaptor protein STING, which is localized on the endoplasmic reticulum (ER) membrane. Ligand binding induces STING to oligomerize and translocate from the ER to the Golgi apparatus, where it recruits and activates TANK-binding kinase 1 (TBK1) [99, 100]. TBK1 phosphorylates IRF3 (interferon regulatory factor 3), which then dimerizes and translocates to the nucleus to induce transcription of type I interferons (IFN-α and IFN-β) and pro-inflammatory cytokines (Fig. 2) [98, 101].

In the CNS, both neurons and glial cells express cGAS and STING, and their activation by mtDNA has been directly implicated in neuroinflammation and NDDs [12, 93]. For instance, in AD mice, mitochondrial damage increases mtDNA leakage into the cytosol, where it engages cGAS and activates STING, driving microglial secretion of type I interferons and pro-inflammatory cytokines that contribute to neuronal dysfunction [93]. In PD, impaired mitophagy due to *PINK1* or *Parkin* mutations results in the accumulation of damaged, ROS-producing mitochondria with mtDNA leakage, triggering cGAS–STING signaling and promoting dopaminergic neuron degeneration [102, 103]. Importantly, beyond neurons, microglia are particularly sensitive to this pathway. Their activation sustains chronic inflammation, recruits peripheral immune cells, and amplifies neuronal injury. While transient activation of cGAS–STING is protective against pathogens, chronic or dysregulated activation by endogenous mtDNA in aging or neurodegeneration becomes maladaptive.

In addition to cGAS–STING, recent evidence indicates that Z-DNA binding protein 1 (ZBP1) can act as an innate immune sensor for cytosolic Z-DNA, a structural form that can arise from damaged mtDNA. Upon recognition of Z-DNA, ZBP1 can activate downstream signaling pathways that overlap with and amplify cGAS–STING–mediated inflammatory responses, including type I interferon and inflammasome activation. This parallel sensing mechanism suggests that mtDNA released from dysfunctional mitochondria can engage multiple cytosolic DNA sensors, thereby enhancing microglial activation and sustaining neuroinflammation. Acknowledging ZBP1 expands our understanding of how aberrant mtDNA contributes to innate immune signaling in NDDs and highlights additional potential targets for therapeutic modulation of sterile inflammation [104].

Therapeutically, targeting this pathway is now being actively explored. Pharmacological inhibitors of cGAS and STING, as well as strategies to neutralize type I interferon signaling, have shown benefits in reducing neuroinflammation and preserving neuronal function in preclinical. Together, these findings underscore that the cGAS–STING pathway is not only a molecular sentinel for pathogen detection but also a critical driver linking mitochondrial stress and mtDNA release to chronic neuroinflammation in NDDs. Understanding this mechanism has opened new opportunities to modulate inflammation and protect neurons in disorders like AD, PD, and HD.

### NLRP3 inflammasome activation—cytosolic danger sensing

NLRP3 inflammasome is a well-characterized multiprotein complex that serves as a critical intracellular sensor of diverse DAMPs and pathogen**-**associated molecular patterns (PAMPs) [105, 106]. Initially discovered in the context of autoinflammatory syndromes, the NLRP3 inflammasome has now been recognized as a central mediator of sterile inflammation in NDDs. Mechanistically, cytosolic mtDNA is a potent activator of NLRP3, providing a direct molecular link between mitochondrial dysfunction and chronic inflammation in the brain [42, 107].

**Fig. 3:**
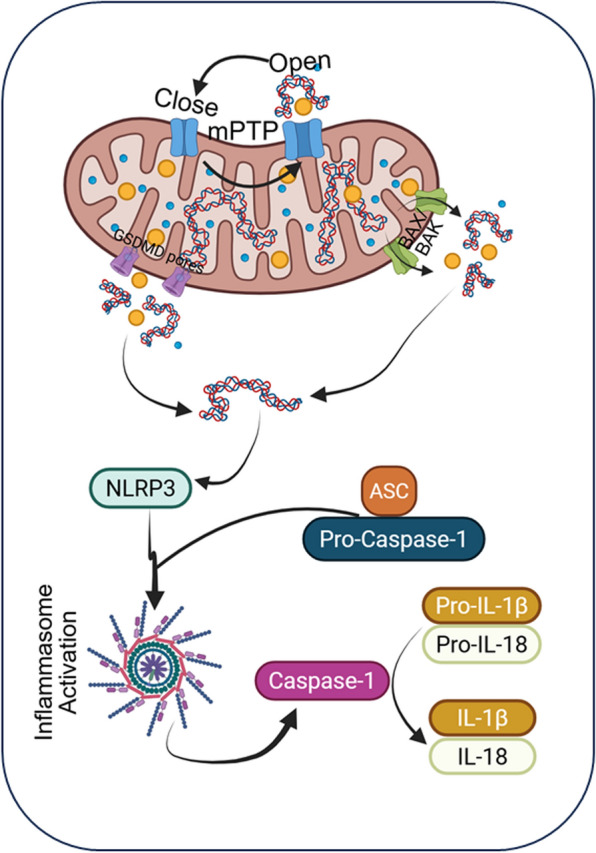
Diagram depicting the mechanisms by which mitochondrial dysfunction and mitochondrial DNA (mtDNA) release promote activation of the NLRP3 inflammasome. Cellular stress leads to mitochondrial damage and the release of oxidized mtDNA into the cytosol. Cytosolic mtDNA acts as a DAMP that facilitates assembly of the NLRP3 inflammasome complex, composed of NLRP3, the adaptor protein ASC, and pro-caspase-1. Inflammasome assembly results in caspase-1 activation, leading to cleavage and maturation of the proinflammatory cytokines IL-1β and IL-18. Sustained mtDNA-driven NLRP3 activation contributes to chronic neuroinflammation and neuronal injury in neurodegenerative disorders

Under stress conditions, such as oxidative injury, mPTP opening, or impaired mitophagy, damaged mitochondria release oxidized mtDNA into the cytosol. This mtDNA can directly bind to and activate NLRP3, triggering its conformational change and oligomerization [223]. Activated NLRP3 recruits the adaptor protein apoptosis-associated speck-like protein containing a CARD (ASC) through pyrin domain interactions. ASC then nucleates the clustering of pro-caspase-1, forming a large supramolecular assembly known as the ASC speck [108, 109]. This close proximity facilitates autocatalytic activation of caspase-1, which is the key effector protease of the inflammasome complex. Activated caspase-1 processes the precursor cytokines pro-IL-1β and pro-IL-18 into their mature, bioactive forms [109]. These cytokines are subsequently secreted and act as potent amplifiers of inflammation: IL-1β promotes the expression of additional inflammatory mediators and adhesion molecules, while IL-18 synergizes to enhance IFN-γ production and immune cell recruitment (Fig. 3) [110]. Beyond cytokine maturation, caspase-1 also induces pyroptosis, a lytic and inflammatory form of programmed cell death that further releases intracellular DAMP, amplifying the inflammatory cascade [87].

In the brain, the NLRP3 inflammasome is primarily expressed in microglia. Activation of NLRP3 by mtDNA released from stressed or damaged neurons leads to microglial secretion of IL-1β and IL-18, establishing a feed-forward loop of neuroinflammation [111]. This process has been directly implicated in the pathogenesis of several NDDs. For example, in AD, Aβ aggregates induce mitochondrial dysfunction and ROS production, leading to mtDNA leakage and NLRP3 activation in microglia [111, 112]. The resulting inflammatory environment contributes to synaptic loss and cognitive decline. In PD, α-syn aggregates also stimulate NLRP3 activation through mitochondrial stress and mtDNA release, exacerbating dopaminergic neuron degeneration [113, 114]. Similar mechanisms are proposed in HD, where mHTT induces mitochondrial fragmentation and oxidative damage, promoting NLRP3-driven inflammation [115].

Historically, these discoveries have reframed the NLRP3 inflammasome not merely as a pathogen sensor, but as a central mediator of sterile, mtDNA-driven neuroinflammation. Therapeutically, pharmacological inhibitors targeting NLRP3 (e.g., MCC950), caspase-1, or IL-1β signaling have shown promise in preclinical neurodegenerative models by reducing neuroinflammation and preserving neuronal function [116–122].

In summary, the NLRP3 inflammasome detects danger signals, including cytosolic mtDNA released from damaged mitochondria, and orchestrates a powerful inflammatory response through caspase-1 activation, cytokine maturation, and pyroptosis. In NDDs, chronic mtDNA-driven NLRP3 activation in microglia fuels sustained neuroinflammation, linking mitochondrial dysfunction directly to neuronal injury and disease progression.

### TLR9—endosomal sensing of CpG DNA

**Fig. 4:**
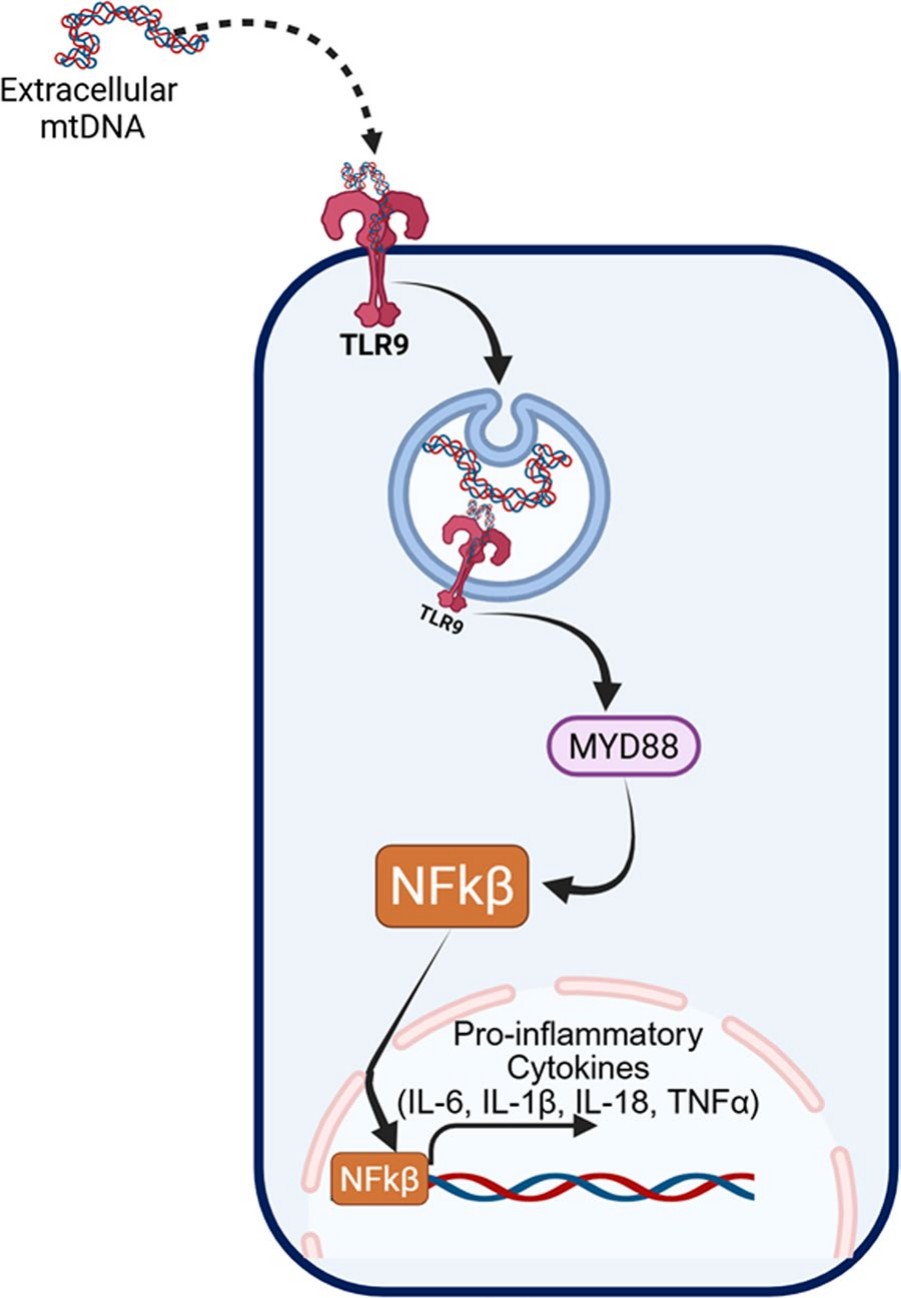
Schematic illustrating how mtDNA released from damaged mitochondria activates TLR-dependent inflammatory signaling. Extracellular or endolysosomal mtDNA is recognized primarily by TLR9 within endosomes of microglia, astrocytes, and infiltrating immune cells. TLR9 engagement triggers MyD88-dependent signaling cascades, leading to the activation of NF-κB and induction of proinflammatory cytokines and chemokines. This mtDNA–TLR axis amplifies innate immune responses, promotes glial activation, and contributes to sustained neuroinflammation and neuronal dysfunction in neurodegenerative and neuroinflammatory conditions

TLR9 is a specialized PRR that evolved to detect unmethylated CpG motifs, which are abundant in bacterial DNA and mtDNA but relatively rare and typically methylated in nuclear DNA [123, 124]. Once released, mtDNA can be internalized into endolysosomal compartments through processes like phagocytosis, autophagy, or uptake of EVs [125, 126]). Within the acidified endosomes, TLR9 directly binds these CpG-rich mtDNA fragments, initiating an innate immune response. Mechanistically, ligand engagement causes TLR9 to recruit the adaptor protein myeloid differentiation primary response 88, via its Toll/IL-1 receptor domain [127]. This interaction triggers the assembly of a signaling complex that includes interleukin-1 receptor-associated kinases and TNF receptor-associated factor 6, leading to the activation of nuclear factor kappa B (NF-κB) and mitogen-activated protein kinases (MAPKs) such as p38 and JNK [128]. These transcription factors translocate to the nucleus, driving the expression of pro-inflammatory cytokines, including TNF-α, IL-6, and IL-1β (Fig. 4) [128].

TLR9 is functionally expressed in microglia and astrocytes, which serve as the brain’s primary immune effector and supporting glial cells, respectively [129]. Activation of TLR9 in these cells by mtDNA released from injured neurons amplifies local inflammation. Microglial TLR9 stimulation increases production of pro-inflammatory cytokines and chemokines, while astrocytic activation contributes to gliosis and release of neurotoxic mediators. In PD and HD, similar processes have been proposed, where mitochondrial dysfunction and defective mitophagy increase mtDNA availability, fueling chronic TLR9-driven inflammation that exacerbates neuronal degeneration. Historically, TLR9 was first recognized for its antiviral and antibacterial roles, sensing pathogen DNA within endosomes [123]. Its role in sterile inflammation, driven by self-DNA such as mtDNA, was later established through studies showing that oxidatively damaged mtDNA can activate TLR9 and trigger inflammation even in the absence of infection [124]. This discovery highlighted how mtDNA, because of its bacterial ancestry, can inadvertently activate ancient immune pathways designed to detect pathogens. Therapeutically, targeting TLR9 or its downstream effectors holds potential to dampen chronic inflammation in NDDs. Preclinical studies using TLR9 antagonists or genetic deletion have shown attenuation of neuroinflammation and improved neuronal survival in models of CNS injury and disease [130].

### Other potential mtDNA sensing pathways

Beyond the well-characterized cGAS–STING pathway, accumulating evidence indicates that additional cytosolic DNA sensors may recognize mtDNA and contribute to innate immune activation, although their specific roles within the CNS remain incompletely understood. One notable sensor is absent in melanoma 2 (AIM2), which belongs to the AIM2-like receptor (ALR) family [131]. AIM2 directly binds cytosolic dsDNA through its HIN-200 domain, independent of sequence, and assembles an inflammasome complex via its pyrin domain that recruits ASC and activates caspase-1. This activation leads to the maturation of IL-1β and IL-18 and can trigger pyroptosis[131, 132]. Although most studies have focused on AIM2 sensing of nuclear or pathogen-derived DNA, recent work suggests that mtDNA released during apoptosis or mitochondrial damage can also activate the AIM2 inflammasome, amplifying inflammation [42]. In the CNS, AIM2 expression has been detected in microglia and astrocytes, and AIM2 activation has been linked to neuroinflammatory responses and cognitive deficits in experimental models of neurodegeneration and traumatic brain injury.

In addition, mtDNA can interact indirectly with the mitochondrial antiviral-signaling protein (MAVS), which is anchored on the OMM and classically activated by viral RNA sensors such as RIG-I and MDA5 [133]. Recent work indicates that oxidative stress or mitochondrial damage can promote MAVS signaling, enhancing the expression of type I interferons and pro-inflammatory cytokines [134, 135]. Although traditionally associated with antiviral responses, MAVS signaling is increasingly recognized as a modulator of sterile inflammation, including in the brain [136].

Collectively, these emerging pathways suggest that mtDNA serves as a versatile DAMP capable of engaging multiple cytosolic sensors beyond cGAS. The activation of AIM2 and MAVS by mtDNA could act in parallel or synergistically with cGAS–STING and NLRP3 inflammasome pathways, amplifying innate immune responses and neuroinflammation. Future studies are needed to dissect the cell-type specificity, temporal dynamics, and pathological consequences of these alternative DNA sensors in NDDs.

## Neuroinflammation and mtDNA

Neuroinflammation is a key process in the CNS, playing a vital role in both maintaining brain health and contributing to disease when dysregulated [137]. The main cells involved in neuroinflammation are microglia, astrocytes, and peripheral immune cells. Microglia are the resident immune cells in the CNS, performing crucial tasks in monitoring and maintaining neuronal homeostasis [138]. Microglia are involved in synaptic pruning, debris clearance, and supporting neuronal health in their resting state [138, 139]. However, when activated in response to injury, infection, or disease, microglia can change into a pro-inflammatory state, producing a wide range of inflammatory molecules such as cytokines (e.g., TNF-α, IL-1β), ROS, and proteases, all of which contribute to tissue damage and neurodegeneration [140]. Astrocytes, the most abundant glial cells in the brain, also play an essential role in neuroinflammation [141]. Under normal conditions, astrocytes help maintain the blood–brain barrier, provide metabolic support to neurons, and regulate synaptic function [142]. However, astrocytes can become reactive and secrete pro-inflammatory molecules in response to inflammation or injury, amplifying the inflammatory response [141]. These reactive astrocytes may further exacerbate neuronal damage, particularly when neuroinflammation persists over time. Peripheral immune cells, such as macrophages and T lymphocytes, can infiltrate the CNS during chronic inflammation. Normally confined to the periphery, these immune cells migrate into the brain in conditions like multiple sclerosis, AD, and PD, where they contribute to the inflammatory cascade [143]. This infiltration can intensify the neuroinflammatory response, further driving neurodegeneration [143].

The immune response in the CNS is highly complex and needs to be finely balanced. On one hand, microglia and astrocytes can mount protective, anti-inflammatory responses, which aid in tissue repair, clearance of damaged cells, and resolution of inflammation [144]. Anti-inflammatory microglia release factors that promote neuronal survival and tissue healing, effectively dampening the inflammatory response once the threat has been removed [144]. The shift from anti-inflammatory to pro-inflammatory microglial activation is a key factor in the transition from protective to harmful inflammation. The imbalance between these pro-inflammatory and anti-inflammatory responses is a central feature in conditions like AD, PD, and HD, where chronic neuroinflammation exacerbates neuronal injury and accelerates disease progression [145]. Furthermore, chronic neuroinflammation is self-perpetuating, creating a vicious cycle where neurodegeneration and inflammation feed into each other. The presence of inflammatory mediators can induce further activation of microglia and astrocytes, which in turn release additional inflammatory factors, leading to even greater neuronal damage. mtDNA itself can act as a transferable inflammatory signal that drives non cell-autonomous propagation of neurodegeneration. Under conditions of mitochondrial stress or impaired mitophagy, mtDNA can be released into the cytosol or the extracellular space. Once in these compartments, mtDNA may be taken up by neighboring neurons, microglia, astrocytes, and, if it enters the bloodstream, by peripheral immune cells, including T cells. mtDNA internalization, or receptor-mediated activation of immune response as DAMP, triggers sterile inflammation and amplifies cytokine and type I interferon signaling, thereby establishing feed-forward loops of mitochondrial dysfunction, immune activation, and secondary mtDNA release. This cycle perpetuates the progression of the disease, ultimately contributing to cognitive decline, motor dysfunction, and other behavioral changes commonly observed in NDDs. Understanding the complex interactions between microglia, astrocytes, and peripheral immune cells is essential for developing targeted therapies that can harness the beneficial aspects of neuroinflammation while mitigating its destructive effects in NDDs.

mtDNA is increasingly recognized as a pivotal mediator linking mitochondrial dysfunction to neuroinflammation in a broad range of neurological disorders. The unique pro-inflammatory potential of mtDNA stems from its bacterial ancestry, allowing it to activate innate immune receptors upon aberrant release into the cytosol or extracellular space. Neuroinflammation, characterized by activation of microglia and astrocytes, and recruitment of peripheral immune cells, plays a dual role in nervous system pathophysiology, where acute responses may be protective, but chronic inflammation leads to progressive neuronal damage and neurodegeneration. This section reviews the roles of mtDNA-driven neuroinflammation in major NDDs and acute CNS injuries, detailing underlying molecular mechanisms, pathological consequences, and therapeutic prospects.

### AD

AD is marked by progressive cognitive decline linked to extracellular Aβ plaques and intraneuronal neurofibrillary tangles composed of hyperphosphorylated tau protein [146]. In addition to these hallmark lesions, mitochondrial dysfunction appears early and consistently in AD pathology [147]. OXPHOS defects, increased production of ROS, and structural abnormalities of mitochondria are often seen in vulnerable brain areas such as the hippocampus and cortex [148]. Postmortem studies have shown higher mtDNA point mutations, large deletions, and lower mtDNA copy numbers in AD patients [149, 150].

There is growing evidence supporting a central role for mtDNA release in driving neuroinflammation and pathology in AD. Multiple human, animal, and cellular studies converge on the view that mitochondrial dysfunction, oxidative stress, and impaired mitophagy lead to leakage of mtDNA into the cytosol and extracellular space, where it activates innate immune sensors and sustains chronic inflammation. Clinical and postmortem human studies show elevated levels of circulating cell-free mtDNA in the cerebrospinal fluid (CSF) and plasma of AD patients, often correlating with cognitive impairment and disease severity. Mechanistic studies in animals have highlighted the role of mtDNA in activating key innate immune pathways. For example, Xie et al. [93] demonstrated in 5 × FAD mice and human AD brains that cytosolic mtDNA engages the cGAS–STING pathway in microglia, driving production of type I interferons and pro-inflammatory cytokines. Similarly, Hou et al. [151] found upregulation of cGAS and STING in APP/PS1 mice; genetic deletion of cGAS reduced neuroinflammation and improved cognitive function. In parallel, Heneka et al. [111] showed that mtDNA released from damaged mitochondria activates the NLRP3 inflammasome, leading to IL-1β production and promoting amyloid pathology in AD mice and human samples. Complementary work by Fang et al. [63] demonstrated that pharmacologically enhancing mitophagy (e.g., with urolithin A) can reduce mtDNA leakage and ameliorate inflammation in APP/PS1 mice. Evidence for TLR9 activation comes from studies like Scholtzova et al. [152], showing that a TLR9 agonist (CpG oligodeoxynucleotides) modulates immune responses and reduces amyloid burden in transgenic AD mouse models, although effects on cognition and plaque clearance appear context-dependent. Roy et al. [224] demonstrated that oxidative stress triggers mtDNA leakage, which activates the cGAS–STING pathway and induces the secretion of pro-inflammatory cytokines in human iPSC-derived neurons. Finally, several reviews [147, 153] have synthesized this body of work, emphasizing how mitochondrial damage and mtDNA release create a vicious cycle: oxidative stress damages mtDNA, increasing its release, which then sustains inflammation and exacerbates amyloid and tau pathology. Additionally, sustained activation of the cGAS–STING signaling impairs microglial phagocytosis of Aβ, promoting Aβ accumulation [151]. This microglial clearance impairment may result from reprogramming microglial function from a homeostatic, phagocytic state to a pro-inflammatory phenotype, which further worsens AD pathology. Overall, these findings highlight the pathogenic role of mtDNA as a neuroinflammatory trigger and its contribution to AD progression.

Pharmacological inhibition of TLR9 or the cGAS–STING DNA-sensing pathway has demonstrated efficacy in mitigating neuroinflammation and cognitive deficits in AD mouse models. For instance, STING inhibition in App^NL‑G‑F^/hTau knock‑in mice normalizes microglial function, reduces type I interferon and NLRP3 activation, decreases Aβ and tau pathology, limits synaptic loss, and preserves memory performance [154, 155]. Complementarily, microglial cGAS deletion significantly curbs early plaque formation and cognitive decline in AD models, underscoring the central role of this innate immune sensor in disease progression [156, 157]. Similarly, treatment with mitochondria‑targeted antioxidants such as Mitoquinone (MitoQ) has shown potent protective effects in preclinical AD settings. In 3 × Tg‑AD mice, 5‑month MitoQ administration prevented the Aβ‑induced oxidative stress, astrogliosis, synaptic loss, and cognitive decline [158]. Although earlier studies in *C. elegans* models suggested MitoQ did not reduce mtDNA oxidative lesions directly, more recent reviews confirm that MitoQ enhances mitochondrial antioxidant defenses, reduces mtROS, suppresses inflammasome activation, and promotes anti-inflammatory microglial polarization, all contributing to reduced mtDNA damage and dampened inflammatory signaling [159]. Together, these findings emphasize the therapeutic potential of targeting mtDNA-mediated innate immune activation, either through TLR9/cGAS–STING pathway inhibition or mitochondria-targeted antioxidant therapy, to attenuate neuroinflammation and cognitive impairment in AD models.

### PD

PD is a progressive neurodegenerative disorder primarily characterized by the selective loss of dopaminergic neurons in the substantia nigra [160]. While the accumulation of α-syn aggregates and mitochondrial dysfunction have long been established as central features of PD pathology, increasing evidence implicates mtDNA as a potent DAMP that links mitochondrial dysfunction to innate immune activation [161]. This connection is largely mediated through the cGAS–STING pathway, which plays a pivotal role in driving neuroinflammation. Genetic mutations in *PINK1* and *Parkin*, two key regulators of mitophagy, are strongly associated with early-onset familial PD [60]. PINK1 accumulates on the OMM in response to mitochondrial damage, recruiting Parkin to ubiquitinate mitochondrial proteins and promote autophagosome-mediated clearance [162]. When this system is impaired, dysfunctional mitochondria accumulate, leading to excessive production of mitochondrial ROS and leakage of mtDNA into the cytosol [163, 164]. Cytosolic mtDNA acts as a ligand for cGAS, initiating a cascade via STING that culminates in production of type I interferons, IL-6, and other pro-inflammatory cytokines. The NLRP3 inflammasome is also activated by mtDNA and mtROS, further contributing to neuroinflammatory cascades [223]. Elevated levels of circulating mtDNA and inflammatory cytokines such as IL-6 and IFN-β have been reported in the CSF and serum of PD patients, particularly those carrying biallelic *PINK1* or *Parkin* mutations [165]. Postmortem analyses of PD brains have revealed increased cytosolic mtDNA and upregulation of cGAS–STING pathway components, especially in dopaminergic neurons of the substantia nigra [166]. Mouse models lacking *Parkin* or *PINK1* develop motor deficits and dopaminergic neuron loss when subjected to mitochondrial stress, particularly in combination with the mutator mouse model, which accumulates mtDNA mutations due to POlG dysfunction [60]. Emerging data indicate that α-syn aggregates not only contribute to mitochondrial damage but also directly or indirectly induce genomic DNA damage. Through oxidative and nitrosative stress, α-syn facilitates the formation of DNA double-strand breaks (DSB), which can also activate cGAS–STING in both neurons and glial cells [167, 168]. This is further supported by the observation of elevated γH2AX, a marker of DSBs, in PD brains. Interestingly, microglia and astrocytes exposed to α-syn fibrils exhibit STING-dependent inflammatory responses, including upregulation of IFN-stimulated genes (ISGs) and increased secretion of IL-1β, IL-6, CXCL10, and CCL20 [169]. These responses can precede neurodegeneration and are significantly ameliorated in STING-knockout models, indicating that cGAS–STING activation plays a pivotal role in α-syn-induced neuroinflammation [170, 171].

The interplay between impaired mitophagy, mtDNA leakage, and cGAS–STING signaling provides several promising therapeutic targets. Enhancing mitophagy (e.g., via urolithin A or nicotinamide riboside) reduces mtDNA burden and inflammation in preclinical models [151]. STING inhibitors, such as H-151 and C-176, have been shown to reduce neuroinflammation and dopaminergic loss in PD mouse models. Likewise, improving mitochondrial dynamics and reducing fission via Drp1 inhibition helps prevent mtDNA fragmentation and release [172, 173]. Additionally, strategies aimed at preserving DNA repair mechanisms, such as enhancing the expression of MRE11 or APE1, could counteract α-syn-induced DNA damage and reduce inflammation [174]. Modulating astrocytic and microglial responses to mtDNA and genomic DAMPs may offer further benefits.

Altogether, mtDNA acts as a molecular switch linking mitochondrial dysfunction to innate immune activation in PD. The cGAS–STING pathway is emerging as a key inflammatory driver in this context, particularly when mitophagy fails to contain mitochondrial damage. Targeting mtDNA release, enhancing its clearance, or inhibiting downstream signaling may open new avenues for disease-modifying therapies in PD. Table 1 summarizes key studies that elucidate the involvement of mtDNA in mediating neuroinflammatory responses in PD.

**Table 1:** Studies linking mtDNA damage to inflammation in PD

Study/year	Model/system	Key findings	Reference
Matsui et al., 2021	Cell models & zebrafish PD models	Cytosolic double-stranded DNA of mitochondrial origin escapes lysosomal degradation and induces cytotoxicity and neurodegeneration	[175]
Borsche et al., 2020	Parkin/PINK1 deficiency models (mouse) & review	Parkin/PINK1 loss impairs mitophagy, leads to mtDNA release and triggers inflammatory responses	[165]
Tresse et al., 2023	Mouse models / brain injection experiments	Damaged mtDNA in brain can be released and, when introduced, elicits PD-like behavioral and pathological changes, linking mtDNA damage to PD phenotypes	[176]
Hinkle et al., 2022	Human tissue + models	STING (downstream of cytosolic DNA sensing) mediates α-syn–driven neuroinflammation and neurodegeneration; implicates DNA-sensing pathways in PD	[170]
Standaert et al., 2022	Microglia / in vitro models	Misfolded α-syn causes DNA damage in microglia and activates cGAS–STING signaling to drive neuroinflammation	[177]
Lowes et al., 2020	CSF from PD patients (PPMI cohort)	Cerebrospinal fluid cell-free mtDNA levels were altered in PD cases and associated with cognitive measures, supporting relevance of mtDNA as a biomarker	[178]
Qi et al., 2023	Peripheral blood cells from PD patients	Increased mtDNA damage in PBMCs from idiopathic PD and *LRRK2* mutation carriers proposes mtDNA damage as a peripheral PD marker	[179]

### HD

HD is a fatal, autosomal-dominant neurodegenerative disorder characterized by progressive motor dysfunction, cognitive decline, and psychiatric disturbances. The disease is caused by an expanded CAG repeat in the *HTT* gene, leading to mHTT expression and widespread neuronal dysfunction and loss, especially in the striatum and cortex [180]. While the genetic basis of HD is well defined, the downstream molecular mechanisms contributing to neuronal death and disease progression remain an area of active research. Among these mechanisms, mitochondrial dysfunction and neuroinflammation have emerged as critical contributors to HD pathogenesis [181, 182]. Recent evidence implicates mtDNA release and activation of cGAS-STING innate immune signaling pathways, in driving neuroinflammation in HD. In HD, mitochondrial abnormalities including impaired respiration, altered calcium handling, and increased oxidative stress have been consistently reported [183, 184]. Dysfunctional mitochondria can undergo permeabilization and fragmentation, leading to the release of mitochondrial components such as mtDNA into the cytoplasm and extracellular space [134].

The release of mtDNA, a pro-inflammatory DAMP, has been shown to activate innate immune responses [33]. mPTP opening, mitochondrial fission regulated by Drp1, and impaired mitophagy are mechanisms contributing to mtDNA release in NDDs, including HD [63, 185]. In HD models, increased mitochondrial fragmentation and impaired clearance have been reported, supporting the possibility of mtDNA leakage as a neuroinflammatory trigger [186]. Notably, we have demonstrated in an HD mouse model that mitochondrial dysfunction leads to enhanced release of mtDNA into the cytosol, which primes innate immune activation via the cGAS–STING pathway. We have shown that the mtDNA-induced cGAS activation in the striatum leads to a robust type I interferon response, exacerbating neuroinflammation and accelerating neurodegeneration. This study provided mechanistic insights linking mitochondrial dysfunction, mtDNA release, and cGAS-STING activation with progressive HD pathology, highlighting a feed-forward cycle of neuroinflammation [12]. cGAS and downstream signaling (STING, TBK1) are upregulated in HD striatal neurons, promoting expression of proinflammatory genes like *Ccl5* and *Cxcl10* [187]. Sun et al. [188] revealed that mHTT disrupts DNA repair by degrading MLH1, causing DNA hyperexcision via Exo1 and cytosolic DNA accumulation. This activates the cGAS–STING-dependent apoptosis and type I interferon responses. Together, these studies link mHTT to innate immune activation through DNA damage and cytosolic DNA sensing.

Chronic activation of the cGAS–STING pathway by mtDNA promotes sustained type I interferon and NF-κB signaling, amplifying neuroinflammation. Neuroinflammation in HD has been linked to exacerbation of neuronal injury via multiple mechanisms, including microglial toxicity, altered synaptic function, and impaired clearance of mutant proteins [189]. By releasing mtDNA and triggering cGAS–STING activation, mitochondrial dysfunction acts as a key upstream driver of neuroimmune responses in HD. These findings open new avenues for therapeutic intervention, such as targeting mitochondrial quality control, mtDNA release, or cGAS-STING signaling to mitigate neuroinflammation and slow disease progression. Modulation of the cGAS-STING pathway holds therapeutic promise. Pharmacological inhibitors of cGAS or STING are under development and have shown efficacy in reducing inflammation in autoimmune and neurodegenerative models. Additionally, improving mitochondrial function, enhancing mitophagy, or preventing mitochondrial fragmentation could reduce mtDNA release and subsequent inflammatory signaling. Inhibiting cGAS–STING signaling with small molecules alleviated neuroinflammation in HD mice, providing a proof-of-concept for translational development. Future studies are needed to precisely delineate the role of the mtDNA–cGAS–STING signaling in HD progression and to explore combinational approaches targeting both mitochondrial health and innate immunity. Advanced techniques such as single-cell RNA sequencing and in vivo imaging will aid in unraveling cell-type-specific dynamics of this pathway in HD. Table 2 summarizes key studies that elucidate the involvement of mtDNA in mediating neuroinflammatory responses in HD.

**Table 2:** Studies linking mtDNA instability and release to inflammation in HD

Study/year	Model/system	Key findings	Reference
Jauhari et al., 2020	HD mice & Q7 vs Q111 striatal cells	Cytosolic mtDNA activates cGAS-STING-IRF3 signaling, driving inflammatory cytokine production; effects reduced by DNase1 or melatonin	[12]
Neueder et al., 2024	Human post-mortem skeletal muscle & cells	Lifelong mutant HTT expression predisposes to mitochondrial network defects and mtDNA instability in post-mitotic tissues	[190]
Beatriz et al., 2022	HD cellular models/tissues	Defective mitochondria-lysosome axis increases release of extracellular EVs that contain mtDNA and mitochondrial proteins in HD	[191]
Sharma et al., 2020	Cellular HD models/mechanistic assays	cGAS (DNA sensor) is a key regulator of inflammatory and autophagy responses in HD models, linking DNA sensing to HD inflammation	[187]
Franco-Iborra et al., 2021	Cellular models (mitophagy assays)	Mutant HTT impairs mitophagy steps, promoting accumulation of damaged mitochondria (mechanistic route for potential mtDNA release)	[192]
Siddiqui et al., 2012	STHdhQ7 vs STHdhQ111 striatal cells	Mutant HTT increases oxidative mtDNA damage and reduces mitochondrial bioenergetics-early biochemical evidence linking mHTT to mtDNA damage	[28]
Hwang et al., 2015	Cellular models/mechanistic	Mutant HTT interacts with GAPDH and impairs GAPDH-induced (micro)mitophagy, promoting persistence of damaged mitochondria	[193]
Sun et al., 2024	Cellular/molecular studies (HD mechanisms)	Recent work on DNA repair/nuclear genome instability in HD that complements mtDNA instability findings (mechanistic context)	[188]

This table summarizes key experimental and clinical studies demonstrating mtDNA damage, instability, or cytosolic/extracellular release in HD models and patient samples. For each study, the experimental model/system (cellular, animal, or human), and key findings are indicated, highlighting the mechanistic link between mitochondrial dysfunction and innate immune activation in HD

## Therapeutic strategies targeting mtDNA-driven neuroinflammation

**Fig. 5:**
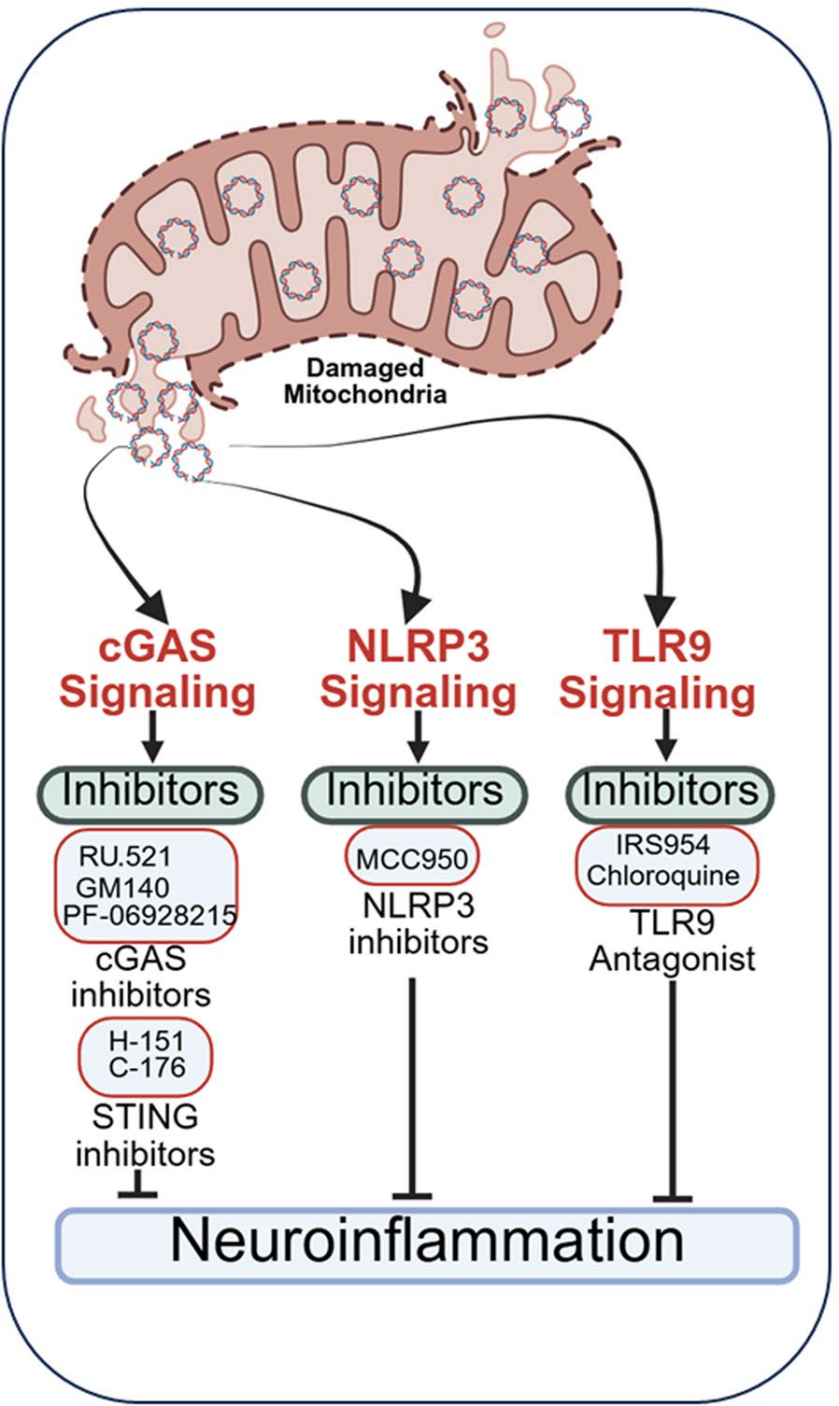
Therapeutic strategies to limit mtDNA-driven neuroinflammation. The strategies include pharmacological inhibition of mtDNA-mediated cGAS–STING signaling using small-molecule inhibitors or antagonists, suppression of NLRP3 inflammasome activation, and blockade of TLR9-mediated inflammatory signaling through targeted compounds. Collectively, these interventions are designed to dampen innate immune activation, reduce glial reactivity, and preserve neuronal function in neurodegenerative and neuroinflammatory disorders

The mtDNA-driven neuroinflammation has emerged as a pivotal pathological mechanism underlying various neurodegenerative and neuroinflammatory disorders [12, 95]. Therapeutic interventions aimed at mitigating this process can be categorized into strategies that reduce mtDNA release, inhibit downstream immune-sensing pathways, promote mtDNA repair, and modulate the metabolic-immune interface (Fig. 5).

### Reducing mtDNA release

The pathological release of mtDNA into the cytosol or extracellular space—often due to mitochondrial membrane permeabilization or dysfunction—is the initiating event driving mtDNA-induced inflammation. Therapeutic approaches focus primarily on stabilizing mitochondrial membranes and enhancing mitophagy to limit mtDNA leakage.

Maintaining mitochondrial membrane integrity is crucial to prevent mtDNA escape. Inhibitors of the mPTP, such as cyclosporin A and sanglifehrin A, have demonstrated efficacy in preserving mitochondrial membrane potential and preventing mtDNA release in neurodegeneration and acute brain injury models [45, 95]. By restricting mPTP opening, these agents reduce mitochondrial swelling and rupture, thereby limiting the release of DAMPs, including mtDNA.

Mitophagy selectively clears damaged mitochondria, thereby preventing the accumulation of mitochondria prone to mtDNA leakage [57]. The PINK1/Parkin pathway is a key regulator of this process. Pharmacological or genetic activation of PINK1 stabilization and Parkin recruitment enhances mitochondrial turnover and reduces cytosolic mtDNA levels [60]. Urolithin A, a natural mitophagy inducer, has shown promise in improving mitochondrial quality control and attenuating neuroinflammation in preclinical models [194]. Other autophagy enhancers, such as spermidine, also indirectly diminish mtDNA release by promoting the clearance of defective mitochondria [195].

### Blocking downstream sensing pathways

Despite a reduction in mtDNA release, residual cytosolic or extracellular mtDNA can activate innate immune receptors to sustain inflammation. Targeting these sensing pathways is therefore a critical therapeutic strategy. Small-molecule inhibitors of cGAS enzymatic activity (e.g., RU.521, G140, PF-06928215) or STING signaling (e.g., H-151, C-176) have shown promise in preclinical neuroinflammation and neurodegeneration models by suppressing excessive type I interferon and cytokine production [12, 196]. TLR9 antagonists, such as synthetic oligodeoxynucleotide antagonists (e.g., IRS-954) and chloroquine, inhibit TLR9 activation and downstream NF-κB signaling, reducing pro-inflammatory cytokine release from microglia and astrocytes [197]. NLRP3 inflammasome inhibitors, such as MCC950, reduce neuroinflammatory cytokines and improve outcomes in neurodegenerative and acute brain injury models [121, 198]. Other indirect inhibitors targeting inflammasome assembly or upstream signaling cascades may also mitigate mtDNA-mediated inflammation [106].

### Promoting mtDNA repair

Repairing damaged mtDNA decreases the pool of immunogenic substrates and preserves mitochondrial function, thereby mitigating neuroinflammation [199]. Enhancing mtDNA repair enzymes, POLG, which serves as the primary mtDNA polymerase, is essential for mtDNA replication and base excision repair of oxidative lesions [200]. Therapeutic strategies aiming to upregulate POLG expression or augment its enzymatic activity may improve mtDNA integrity. These include gene therapy approaches to deliver functional POLG or small molecules that stabilize POLG complexes [201, 202]. Furthermore, boosting other mitochondrial repair enzymes such as mitochondrial uracil-DNA glycosylase (UNG1) and 8-oxoguanine DNA glycosylase (OGG1) is reported to enhance mtDNA repair capacity and reduce the mutation burden [203].

### Modulating the metabolic–immune interface

Mitochondrial metabolism is tightly coupled to innate immune signaling, which may be an additional target to attenuate mtDNA-driven neuroinflammation. Excess mitochondrial ROS causes oxidative damage to mtDNA and triggers its release, activating downstream immune pathways. Mitochondria-targeted antioxidants such as MitoQ, SkQ1, and SS-31 have been developed to selectively scavenge mitochondrial ROS, thereby protecting mtDNA from oxidative lesions and preventing inflammatory activation [204]. Clinical trials evaluating these antioxidants in NDDs have reported safety and initial efficacy, supporting their therapeutic potential. Further, metabolic drugs, metformin, a first-line treatment for type 2 diabetes, exhibits neuroprotective and anti-inflammatory effects via activation of AMP-activated protein kinase, inhibition of mitochondrial complex I, and modulation of mitochondrial metabolism. By reducing mitochondrial ROS production and enhancing bioenergetic efficiency, metformin decreases mtDNA damage and attenuates mtDNA-mediated activation of innate immune sensors such as the cGAS–STING pathway and inflammasomes [205]. Emerging evidence also suggests that metformin promotes mitophagy and mitochondrial biogenesis, further limiting mtDNA release and neuroinflammation [206–208]. We have summarized current mitochondria-targeted therapeutic developments in Table 3.

**Table 3:** Mitochondria-targeted therapeutic strategies for NDDs

Therapeutics	Mechanism	Indication(s)	Stage of development	Reference
Elamipretide (SS-31 / MTP-131)	Mitochondrial membrane/cardiolipin stabilization; reduces oxidative stress	PD, AD models; approved for Barth syndrome	Preclinical for NDDs; FDA-approved for Barth syndrome (Sept 2025)	[209]
Mitophagy enhancers (e.g., PINK1 activator ABBV-1088)	Enhance mitophagy via PINK1	PD, NDDs	Phase 1 (NCT06414798)	[210]
Mitophagy enhancers (e.g., USP30 inhibitors MTX115325, FT385)	Inhibit USP30 to boost mitophagy	PD, AD	Early clinical/Phase I targeted	[211]
cGAS–STING inhibitors (e.g., H-151 and others)	Inhibit cytosolic DNA sensing & type I IFN inflammation	Neuroinflammatory NDDs	Preclinical	[212]
NLRP3 inflammasome inhibitors	Suppress inflammasome activation & IL-1β/IL-18	PD, AD models & other inflammatory conditions	Early clinical/preclinical	[213]
Selnoflast (RO7486967)	NLRP3 inflammasome inhibitor	Early PD neuroinflammation	Phase Ib PD safety	[214]
Nicotinamide Riboside (NR)	NAD^+^ precursor; supports mitochondrial metabolism	PD	Phase I trial (1000 mg × 30 days)	[215]
Coenzyme Q10 & derivatives (MitoQ, SkQ1)	Mitochondrial antioxidants; reduce ROS	AD/PD/HD models	Clinical/Preclinical	[216]
Melatonin/Astaxanthin	mPTP inhibition; antioxidant	AD/MCI	Clinical (Phase II/III)	[217, 218]
Hydralazine	Autophagy activation; NRF2 antioxidant regulation	AD	Phase III	[219]
Glutathione (GlyNAC)	Antioxidant support	AD/MCI	Clinical	[225]
Pridopidine	Sigma-1 receptor modulator; mitochondrial support	HD, PD, AD models	Preclinical/early clinical interest	[220]
Metabolic modulators (e.g., terazosin, deferiprone, sulforaphane)	Mito metabolism/antioxidant pathways	PD	Phase II or early	[221]

This table summarizes current and emerging therapeutic approaches aimed at mitigating mitochondrial dysfunction in NDDs. For each intervention, the molecular target, proposed mechanism, disease context, and stage of development (preclinical or clinical) are indicated

## Challenges and future perspectives

mtDNA has emerged as a critical driver of neuroinflammation, linking mitochondrial dysfunction to innate immune activation across diverse neurological disorders. However, translating this growing knowledge into reliable diagnostics and effective therapies remains challenging. Accurate detection and quantification of mtDNA release in the CNS are hindered by several factors. Cytosolic and extracellular mtDNA are often present at low levels and rapidly degraded by nucleases, complicating their measurement. Moreover, distinguishing mtDNA from nuclear mitochondrial DNA segments requires highly specific assays. While PCR-based methods are widely used, they lack sensitivity and specificity; droplet digital PCR offers improvements but lacks standardization. In biofluids like CSF and plasma, fragmented cell-free DNA further complicates interpretation, underscoring the need for enhanced sample processing and mtDNA-targeted probes. These constraints limit the development of robust mtDNA biomarkers and impede therapeutic monitoring. mtDNA-driven neuroinflammation varies widely by cell type and disease context. Microglia predominantly utilize cGAS–STING and inflammasome pathways, whereas astrocytes may engage TLR9 signaling. The temporal dynamics of mtDNA release and immune responses differ between acute injuries (e.g., stroke, traumatic brain injury) and chronic NDDs. Furthermore, brain region-specific vulnerabilities and aging influence mtDNA-mediated inflammation. Current experimental models often fail to capture this heterogeneity, highlighting the need for cell-type-specific, spatially resolved, and longitudinal studies.

Recent advances offer powerful tools to overcome existing challenges. Single-cell RNA sequencing combined with spatial transcriptomics enables high-resolution mapping of mtDNA sensing and inflammatory pathways within distinct cell populations in situ. Advanced imaging techniques, including super-resolution microscopy and mitochondrial-targeted fluorescent reporters, allow real-time visualization of mtDNA release and mitochondrial dynamics in vivo. Integration with proteomics, metabolomics, and epigenomics provides a comprehensive multi-omics perspective. Artificial intelligence and computational modeling can synthesize these datasets to identify novel regulatory networks and therapeutic targets.

## Conclusion

mtDNA functions as a crucial regulator of neuroinflammation, bridging mitochondrial dysfunction with innate immune activation through DAMP/PRR such as cGAS–STING, TLR9, and inflammasomes. This dual role of mtDNA, initiator of acute responses and perpetuator of chronic inflammation, links metabolic distress to progressive neuronal injury in aging and NDDs. Despite advances elucidating mtDNA release, sensing, and downstream signaling, critical gaps remain, particularly concerning cell-type-specific dynamics and integration with other pathological processes. Improved detection technologies and multi-omics approaches promise to resolve these complexities and accelerate biomarker discovery and therapeutic development. Therapeutic strategies targeting mitochondrial integrity, mtDNA repair, and selective inhibition of mtDNA sensing pathways hold promise to mitigate neuroinflammation and neurodegeneration. Metabolic modulators such as antioxidants, metformin, and melatonin also show potential by modulating mitochondrial and immune pathways. Realizing these advances clinically demands multidisciplinary collaboration spanning mitochondrial biology, immunology, neuroscience, and clinical research. Ultimately, harnessing mtDNA’s central role could transform diagnosis, prognosis, and treatment of devastating neurological diseases.

## Data Availability

Not Applicable.

## Abbreviations

mtDNA: Mitochondrial DNA
cGAS: Cyclic GMP–AMP synthase
STING: Stimulator of interferon genes
TLR: Toll-like receptors
DAMP: Damage-associated molecular pattern
NDDs: Neurodegenerative diseases
AD: Alzheimer’s disease
PD: Parkinson’s disease
HD: Huntington’s disease
ALS: Amyotrophic lateral sclerosis
OXPHOS: Oxidative phosphorylation
ROS: Reactive oxygen species
ETC: Electron transport chain
POLG: Polymerase gamma
mPTP: Mitochondrial permeability transition pore
OMM: Outer mitochondrial membrane
CypD: Cyclophilin D
MOMP: Mitochondrial outer membrane permeabilization
PRR: Pattern recognition receptors
EV: Extracellular vehicles
cGAMP: Cyclic GMP–AMP
TBK1: TANK-binding kinase 1
IRF3: Interferon regulatory factor
NLRP3: NOD-, LRR-, and pyrin domain-containing protein 3
PAMP: Pathogen**-**associated molecular patterns
ASC: Apoptosis-associated speck-like protein containing a CARD
NF-κβ: Nuclear factor kappa beta
MAPKs: Mitogen-activated protein kinases
TNF-α: Tumor necrosis factor-alpha
